# Specialization in sports: A theoretical approach

**DOI:** 10.1371/journal.pone.0250722

**Published:** 2021-05-05

**Authors:** Anne C. Wunderlich, Florian Follert, Frank Daumann

**Affiliations:** 1 Swiss Federal Institute for Forest, Snow and Landscape Research (WSL), Birmensdorf, Switzerland; 2 Seeburg Castle University, Seekirchen, Salzburg, Austria; 3 Institute of Sports Science, FSU Jena, Jena, Germany; Universitat de Barcelona, SPAIN

## Abstract

Nations have been competing in sporting competitions for centuries. Therefore, explaining the success of different countries has a long history in sports science. At first, researchers tried to explain success patterns with the help of divergent geographical factors. Later, literature included other determinants on the macro-level which provide evidence that especially the GDP as a proxy for the prosperity of a country has a significant impact on success in sports. Within this broader field of research, also specialization patterns in sports developed into an important topic of research. In line with the literature on factors which lead to (national) success, so far, the discussion mostly concentrates on determinants on a macro-level. We identify the problem that different specialization patterns can be observed in countries that have similar factors on the macro-level, as well. There seems to be a research gap concerning the influencing factors on a meso-level. As a result, the aim of this paper is to show which determinants on the meso-level can affect sports specialization patterns. We provide a model based on the findings of lobbying theory that explains not only different specialization patterns between, e.g., Europe and Africa, but also different specialization patterns within a continent and dissimilar patterns of countries with a similar macro-level can be understood. Overall, our paper contributes to the discussion on specialization in elite sports from an economic perspective, so that future research can build on our work, in particular concerning empirical tests of our approach.

## 1 Introduction

*“All kinds of being in these stretches of land were capable by nature of special feats of en-deavor. […] Running is certainly in the blood of every Finn. When you see the clear, deep green forests, the wide open luxuriant plains […], the heights covered by massive clusters of trees and never ending light blue to the horizon with the lakes merging with the sky, one is overcome by an involuntary feeling of elation and because you don’t have wings, you want to run.”* ([1],p. 61)

Nations have been competing in sporting competitions for centuries. Sporting success often has an important effect on the political level. Governments regularly bask in the success of their athletes and the state promotes elite sport. Especially during the Cold War, the medal table was a symbol of the competition between a socialist and a capitalist economic model (e.g., [2]). During the 1920s and 1930s, success in international sports competition was mostly exclusively explained by different geographical conditions that affect different countries, which may be irritating from today’s perspective of a multicultural and globalized world, but does not seem entirely unsuitable for the exclusive purpose of the topic under consideration here. Therefore, the success of African American runners was often explained by the physical adaptation over history to the environment in which their ancestors lived earlier [3]. For instance, the success of Austrian ski racers could be explained by the alps, where generations of humans had to learn ski to get from the mountain to the village (on success in alpine ski racing [4]). Even years later, in the nineties of the last century, it is still emphasized that the primary reason for different sports must be seen in divergent geographical factors [5]. Not only sporting success but also the participation in Olympic Games was explained with the help of different climate conditions. Jokl (1964) concludes that the participation rates during the Olympic Games 1952 is dependent on areas with cool and warm, warm, as well as cool temperature zones [6]. Asides of the geographic-variations argument in the “production” of athletes, historical, and cultural or social factors were not discussed [1]. Today, the research concerning factors that determine sporting success is more diverse and also considers other factors than geographical determinants on the macro-level. Other influencing factors besides the geographical determinants are cultural, social, demographic or political factors, especially the role of the government (e.g., [7]). An important discussion in the political analysis of sporting success deals with the conditions within a free society and in a totalitarian system (e.g., [8]). Furthermore, the country- or region-specific sporting success is closely linked to the specialization that is well known as “division of labor” from economic theory in the more general case (e.g., [9]). Specialization means that states focus the relevant resources on a limited number of sports—or even just one. An optimal specialization to specific sports is based on the knowledge that specific factors determine athletic success and that a specific set of these factors respectively a specific factor endowment like e.g., a specific geographical endowment or a specific economical endowment favours the success in a specific sports. Given these circumstances an optimal specialization means that states put their relevant available resources in those sports disciplines in which they have a favourable set of factor endowment.

Surprisingly, these specialization patterns are not only different between continents, but also within them. E.g., Britain counts as the cradle of sports from where sports diffused into other developed (and later least developed) countries [1]. But even countries with similar (geographic or climate) conditions, as it is the case within Europe, are successful and specialized in different disciplines. For instance, track and field was originated in Britain, but by 1930 this sport was exercised especially in Germany and in the Scandinavian countries [1].

Nevertheless, many studies which try to determine the factors of specialization patterns exclusively make use of determinants on the macro-level and those determinants long appear to coincide within Europe. And this is where the aim of this paper begins: We want to explain specialization patterns within countries that have similar conditions (like e.g., weather) on the macro-level but still different specialization patterns. For this reason we provide a two-country model that is similar to those which are common in lobbying theory with two sports systems and two different disciplines a country can specialize in (see e.g., [10]). We reveal under which circumstances the countries specialize in different disciplines although they rely on completely similar factors on the macro-level.

The remainder of the paper is structured as follows: First we would like to give a short overview on prior literature concerning the factors which have an effect on success in sports and theories of specialization in sports as well as factors which lead to specialization in sports. Then we will present the model (chapter 3) that gives rise to specific sports policy implications, which will be discussed in the fourth chapter. At the same time, the limitations of the model are clarified in this chapter. A brief conclusion is drawn in the last chapter.

## 2 Previous literature: Specialization in sports

Division of labor and specialization can be attributed to various factors. A significant influence is often seen e.g., in climatic conditions. It is easy to see that e.g., wine can be better produced in warm regions. Every economic student therefore knows the Ricardian example of division of labor between Portugal and England within international trade from their textbooks. With respect to the topic of national success and specialization in sports, apart from earlier research that concentrated on geographical factors to explain international success [3] or [5] as mentioned above. More recent studies focus on other factors, which determine the national success within a country (e.g., [7, 8, 11–23]). Variables that are tested frequently are e.g., the population size (e.g., [16]) or income per capita (e.g., [18]). Even if countries have similar goals concerning sporting success, the amount of the national sport budget as well as the collective effort within a country should influence the success in the competition with other countries [24]. In particular, there seems to be empirical evidence that the international success is correlated with a range of economic, demographic, social and political variables. Bernard and Busse (2004) use panel data from the Summer Olympics of the last 30 years and resume a positive correlation between the national GDP and the medals this nation can win in international competition. They state that an average country could raise their total account of medals by 1%-1.5% if such a country could double its GDP [16]. Other significant factors are the size of the population as well as the political system (former soviet countries win more, on East Germany as an “optimizing dictatorship” see [2]. Gelade and Dobson (2007) as well as Hoffmann, Lee and Ramasamy (2002) identify important variables which determine the winning probability in soccer [15, 25]. They conclude similar to Bernard and Busse (2004) that the (per capita) wealth of a nation determines international sporting success (measured by the FIFA ranking of a nation). Furthermore, the participation in soccer (and therefore the talent pool) and the length of football tradition within a country, the percentage of expatriate players in a national team and climatic conditions seem to play a crucial role [16]. However, other studies find no evidence that climatic origin correlates to national Olympic success but conclude that a revealed comparative advantage (RCA) can be explained by the covariation of population, gross domestic product and the socialist effect [26].

Despite the wide range of influencing factors, it is not yet clearly elaborated which factors are leading mostly to international sporting success [27]. Especially the role of the policy factors concerning success is not totally understood so far. That policy factors are important to success in sports is clear: E.g., a study which aimed to examine this relationship interviewed 140 Flemish sportsmen, 119 coaches and 26 performance directors. The authors come to the result that eight out of the ten important factors which lead to sporting success can be categorized as sport policy factors. However, the study does not provide any empirical evidence which factors are decisive concerning success in sports. Furthermore, the factors the authors defined as having an effect were defined arbitrarily by the authors, i.e. using a practitioners’ subjective theory [27].

These aforementioned studies also show that determinants of success differ within disciplines: E.g., Switzerland obviously offers a better geographical environment to produce winter sport athletes than any country in Africa. In consideration of these findings, Tcha and Pershin (2003) as well as DuBois and Heyndels (2012) transfer the Ricardian idea of international specialization in trade to sports [24, 28]. Similar to David Ricardo they assume that countries concentrate on those sports they have a comparative advantage in, as nations have different amounts of resources in capital and labor and as there are disciplines that are more capital-intensive (e.g., yachting) and others that are more labor-intensive (like, e.g., long-distance running). Overall, a country tries to allocate scarce resources in the most efficient way. Therefore—within a simple two-factor (capital and labor) model—, it is comprehensible that e.g., a country in Africa which is relatively poorly endowed with capital would always concentrate on a relatively labor-intensive sport, such as running. Due to different relative factor endowments, the performance in some disciplines is relatively better than in other disciplines. This leads to a RCA in those disciplines [24]. Tcha and Pershin (2003) use data of 66 countries in six groups of sports (swimming, athletics, weight games, ball games, gymnastics and other disciplines) for the Summer Olympics. With the help of the Balassa-indicator which is used to measure RCA they show that a nation can be ranked first in total medals in a discipline but at the same time be ranked less high in the RCA ranking (in comparison to other countries): E.g., the United States are ranked first in total medals in athletics (4th per capita medals) but 20th in RCA. In contrast Ethiopia is ranked 13th in total medals but first in RCA. In a next step, Tcha and Pershin (2003) analyze which variables play a crucial role in having a RCA [24]. They use economic, political, geographical as well as physical (ethnic) data as explanatory variables and find clear evidence for specialization in international sport: Based on their findings we can recognize that e.g., nations with a RCA in athletics have a relatively greater landmass, a higher altitude, a higher GDP per capita and fewer coastlines. Furthermore, being an African country turns out to be significant and positive in athletics. In contrast, former Soviet or East European countries have a RCA in gymnastics. Overall, the authors show that using data that is based only on overall or per capita performance (such as total medals won or medals per capita) reveals other results than using RCA: Some variables only become significant when using the RCA index (such as land mass or altitude). Therefore, they conclude that using knowledge on specialization patterns or comparative advantage in sports competition is required when analyzing such data.

In order to examine specialization patterns in sports, DuBois and Heyndels (2012) aggregate one of the groups of sports of the former study of Tcha and Pershin (2003) in their study: athletics. Instead of using the Balassa-indicator, they make use of an index of Revealed Symmetric Comparative Advantage (RSCA), which follows Laursen (2000) who proposed using this index for econometric analysis [29]. They divide athletics in four groups: sprinting and middle-distance running, long distance running, non-running events and race walking. DuBois and Heyndels (2012) analyze data from IAAF rankings of 2005 ([28]). Using almost the same explanatory variables as Tcha and Pershin (2003) their results are quite similar. They also find out that clear specialization patterns in athletics exist. Wealthier (measured by per capita GDP) countries focus on different disciplines in comparison to poorer nations. E.g., Africa and the Caribbean are less specialized in non-running events (and race walking) but more specialized in long distance running (Caribbean has a RCA also for sprinting disciplines). The larger a country the more it is specialized in sprinting and middle distance running and seems to be disadvantaged in non-running events. The authors explain this by the longer travelling distances to sports infrastructure and training facilities. The dummy variables for countries of the former Soviet Union are significant for all running events. This means in reverse: policy matters and seems to play a role to specialization patterns in sports. More precisely, we can conclude from literature that countries seem to invest especially in sports they were traditionally successful in or sports that are culturally important [30]. Medal potential plays a crucial role for funding. E.g., funding for the Olympic Winter Games show that there is a high correlation of public funding with past and present success (see e.g., [31] or [32]). Once the decision on funding has been made, it seems to reinforce itself, establishing a further competitive advantage (e.g., [33]). Furthermore, the so-called Matthew Effect gives a potential explanation to what extent public funding follows success in the first place (see [34] and in sports see [4]).

So far, based on prior literature we know that especially monetary and geographical aspects were verified as being relevant determinants of international sporting success: GDP and population size seems to be the most important factors of the success, while country size and the part of the population which lives in big cities have no significant effect [8]. Strikingly, most studies analyze the relationship between sporting success and factors like economic welfare, population size, the political system or geographical characteristics. The variables that are used in the studies which investigate the relationship between several factors and specialization are similar to the variables that were analyzed as being important for international success in sport events: Tcha and Pershin (2003) as well as DuBois and Heyndels (2012) make use of these macro-level factors to reveal specialization patterns [24, 28].

Nevertheless, the RCA index for several countries shows that although some countries have similar characteristics on the macro-level, they reveal different specialization patterns. An issue that the paper by Tcha and Pershin (2003) cannot give a satisfying answer for, as in their empirical analysis the authors no longer differ between the 66 investigated countries but only between continents. Austria, Belgium, Denmark, France and Germany which are European countries have different RCAs.

Due to the lack of explanatory power of approaches based solely on macro-level factors, research more and more comes to consensus that not only-macro-level factors play a crucial rule to success of a nation [35]. Non macro-level factors are used in [36–39] to explain the success of a nation. For example Digel (2005) discusses several factors leading to sporting success including individual factors like ideological preconditions, Olympic traditions or staff structure while Böhlke (2007) emphasizes the role of the operational communication and management processes, especially the role of coach education systems for the success of the Swedish athletics team and the Norwegian national cross-country skiing team. Truyens et al. (2014) emphasize the importance of structural conditions that determine the management of resources in sports policy based on interviews with coaches and managers in elite sport. They build a list of 98 organizational resources as basis for the development of RCA [38]. Based on this study Truyens et al. (2016) develop a configuration analysis to explain how a specific nation can strategically combine these resources to produce a RCA [39].

De Bosscher et al (2007) classify these other factors that determine overall sporting success with help of a qualitative exploration. Next to the macro-level factors, which were already mentioned, they assign sports policies and politics to factors on the meso-level and genetic qualities as well as the close environment of an athlete (e.g., family, coaches) to factors on the micro-level [27]. As meso-level factors differ not only from continent to continent but also within European countries, we propose to explain the different specialization patterns in sports with the help of policy decisions. The following model could be used as an explanatory approach to understand those determinants on a meso-level.

## 3 The model

As discussed, the shortcoming of the existing literature is that it explains specialization patterns in sports exclusively with help of the relative factor endowments of countries. Literature examines the issue using neoclassical trade theories. The empirical studies show that policy has an influence on specialization patterns and therefore, specialization patterns are not exclusively determined by the factor endowment (macro-level factors) of countries. Factors on the meso-level seem to be decisive to specialization patterns in sports as well. Tcha and Pershin (2003), e.g., use a dummy variable for Asian and African countries but also for countries of the former Soviet Union. Their empirical results show that the former Soviet countries’ dummy plays a significant role for RCA. Consistent with these findings and also with politics (meso-level factors) influencing success, we assume that policy as well as the history of public funding provided to the sport system are important factors and determine specialization patterns in sports. Overall, the government can support sports through subsidies and regulations [40]: Subsidies represent governmental benefits and can be designed directly or indirectly. While direct subsidies are financial or other support in favour of a beneficiary (such as an athlete or a sports organization), indirect subsidies are based on exceptions in tax law (such as income tax exemptions or VAT exceptions for sports organizations or sports events). Direct subsidies can, in turn, take the form of financial benefits or benefits in kind, e.g., free provision of sports facilities [41]. In addition to the subsidies, the government can create redistributive effects by regulating the market. For example, the approval of a joint selling of broadcasting rights can lead to monopoly pricing in this market and thus generate an income distribution at the expense of the broadcasting stations and in favour of the football clubs. Nevertheless, our model assumes that the state acts as unit without federal elements.

The model we use is similar to models used within lobbying theory (see [10] or [42]). We show to what extent political systems influence the specialization in sports. Besides, we can explain why even countries which have a similar background in politics can have different specialization patterns.

The basis for our assumption is that history matters and that the public funding to the sport system were defined in the past and cannot be changed from one day to another. This means that the decisions on the funding to sports has been made in the past but are still in action and dependent on initial conditions. We assume two nations (nation 1 and nation 2) and each country funds its own sport system. This means nation 1 only invests in sport system (I) and nation 2 respectively only funds its own sport system (II). Therefore, the total amount of the funding is expressed as *f*1 and *f*2. We build on a rational choice approach and therefore, both nations aim to maximize their expected utility and choose the amount of funding to the own sport system according to their utility function with stable preferences. Clearly, nations that concentrate their funding on a specific sports discipline can only do so at the expense of governmental funding to another sports discipline. Nations have to choose in which sports they want to concentrate its funding.

Referring to the utility function, it is necessary to note that, as sport systems depend on the existence of other sport systems so they can compete, sports need to be treated as a special case in the entrepreneurial world. Also, the prestige a nation can achieve through sports can only be achieved in competition with other sport systems. Szymanski and Zimbalist (2005) state: “Without opponent no team can produce anything at all.” and hence point out that a sport system is always reliant on another sport system [43]. Due to the necessity of the existence of opponents, we assume that both states derive utility from the existence of both sport systems (I and II) though the states only fund their own sport system. The sport system’s probability to win a competition (*π*) in turn is dependent on the contributions the nations make to their sport system as these contributions determine the Contest Success Function (CSF) of a sport system. In case of no public funding at all from neither the one nor the other state to the own (nor the other) sport system leads to a winning probability of 50% for each sport system. In this case, we assume each sport system has equal opportunities to win a competition. The CSF therefore is defined as:

$$\begin{array}{c} \pi =\{\begin{array}{cc} \frac{f_{1}}{f_{1}+f_{2}} & \text{if}\;f_{1}+f_{2}>0\\ 0.5 & \text{if}\;f_{1}=f_{2}=0 \end{array} \end{array}$$ (1)

The expected utility function of nation j (j = 1, 2) follows subject to the CSF (*π*):

$$\begin{array}{c} U_{j}\left(S_{j}\right)=\pi u_{j}\left(S_{I}\right)+\left(1-\pi \right)u_{j}\left(S_{II}\right)-f_{j} \end{array}$$ (2)

Whereby *uj*(*S*_*I*_) is the utility of nation j (j = 1, 2) derives of sport system I and *f*_*j*_ is the contribution nation j (j = 1, 2) makes to its own sport system. Sport systems are assumed to maximize their utility by maximizing their winning probability:

$$\begin{array}{cc} \text{max}U_{I}=\text{max}\pi \; & \text{and}\;\text{max}U_{II}=\text{max}(1˘\pi ) \end{array}$$ (3)

Sport systems determine simultaneously on which disciplines they promote. However, they rely on decisions made in the past and the patterns of funding cannot be changed fast. Therefore, it is imperative that

$$\begin{array}{cc} S_{I}=\frac{dS_{I}}{dt}=s\frac{d\pi }{dS_{I}}\; & \text{and}\;S_{II}=˘s\frac{d\pi }{dS_{II}} \end{array}$$ (4)

whereby s is the speed with which the current sport policy can be changed. Afterwards the nations contribute the utility-maximizing funding to the sport systems. This is a two-stage game and on the second stage, the nations 1 and 2 decide on the amount of funding. Though nation 1 acquires utility from sport system I and sport system II, the utility derived of sport system I is higher: *u*_1_(*S*_*I*_) > *u*_2_(*S*_*II*_) (respectively for nation 2). Backwards induction leads to the objective functions. First, we define *p*_1_ = *u*_1_(*S*_*I*_) ˘ *u*_1_(*S*_*II*_) and rewrite the utility function of nation 1:

$$\begin{array}{c} U_{1}=\pi u_{1}\left(S_{I}\right)+(1˘\pi )u_{2}\left(S_{II}\right)˘f1=\frac{f_{1}}{f_{1}+f_{2}}p_{1}+u_{1}\left(S_{II}\right)˘f_{1} \end{array}$$ (5)

Respectively, *p*_2_ = *u*_2_(*S*_*II*_) ˘ *u*_2_(*S*_*I*_) leads to

$$\begin{array}{c} U_{2}=\frac{-f_{2}}{f_{1}+f_{2}}p_{2}+u_{2}\left(S_{II}\right)˘f_{2} \end{array}$$ (6)

for nation 2. We now can derive the reaction function $\frac{dU_{1}}{df_{1}}=0$ for nation 1 and $\frac{dU_{2}}{df_{2}}=0$ for nation 2:

$$\begin{array}{c} \frac{dU_{1}}{df_{1}}=\frac{f_{2}}{{\left(f_{1}+f_{2}\right)}^{2}}p_{1}˘1 \end{array}$$ (7)

and,

$$\begin{array}{c} \frac{dU_{2}}{df_{2}}=\frac{f_{1}}{{\left(f_{1}+f_{2}\right)}^{2}}p_{2}˘1. \end{array}$$ (8)

Which together imply then:

$$\begin{array}{c} \pi =\frac{p_{1}}{p_{1}+p_{2}}. \end{array}$$ (9)

Also, the reaction function leads to $f_{1}=p_{1}^{2}p_{2}/{\left(p_{1}+p_{2}\right)}^{2}$ and respectively for 2. Following Ursprung (2002) we assume the convex utility function: *u*_1_ = (*S* ˘ 1)2 and *u*_2_ = *πS*2 with *π* > 0. Using the binominal theorem, we have

$$\begin{array}{c} \pi =\frac{\left(S_{I}+S_{II}-2\right)}{\left(1-\pi \right)\left(S_{I}+S_{II}\right)-2} \end{array}$$

Now, with the starting definition of *S*_*I*_ and *S*_*II*_ and as $\frac{d\pi }{dS}$ is the same for *S*_*I*_ and *S*_*II*_ we have *S*_*I*_ = ˘ *S*_*II*_ < 0. This expression shows that changes in the sport policy can occur in the following way: Nation 1 will reduce *S*_*I*_ whilst nation 2 will increase *S*_*II*_ until (*S*_*I*_, *S*_*II*_) = (0, 1) is reached which means that even if countries start with a situation where both countries concentrate on the same discipline in the long run a specialization on completely different sporting disciplines is possible. This is a situation we can observe in reality as already mentioned in literature (e.g., [24]).

A good example of how sports policy can have an impact on sports success is the GDR and its well-planned approach to elite sport development. The government exerted an enormous influence on its sports system by funding athletics, gymnastics, soccer (only due to its popularity) as well as female swimming and success followed funding. Especially the strategy behind the funding of female swimming was remarkable and based on identifying such sports with less competition.

Some countries on the other hand have a well-established strategy in their funding due to a historical identification of sports with RCA. Examples for such countries are Cuba and boxing, China and table tennis, South Korea and archery or Australia and swimming. For other countries it has been an ongoing challenge to concentrate their resources in those sports (or events) in which they have or consider to have a RCA. Therefore, the strategy often is to bundle resources on sports that were e.g. successful a the Olympic Games: Clearly, sports disciplines with many medals can achieve more funding (see. e.g. funding for male sprinting in the USA) [31].

Even within Europe sport success and funding patterns are very dissimilar. Whilst Germany developed and maintained a clear RCA (within the Olympic Games) in canoeing and rowing, Britain e.g. specializes in cycling and sailing, indicating a strategic funding in these sports [31]. Different success patterns are therefore also due to specializing the funding on a minority of sports and such strategies have become more and more prevalent (see [44] or [45] and [46]).

With help of our model we can provide an explanatory approach for the phenomenon observed that can contribute to a better understanding of sport specialization.

## 4 Discussion, policy implications, and limitations

Sports appears worthy of public support for numerous reasons, not least because of its empirically proven positive influence on life satisfaction (e.g., [47]) of the population. Elite sport success can also have (at least temporary) positive effects on the citizens of a country, e.g., on national pride [48]. Our analysis is based on a rational choice model. Therefore, state action is also subject to budget constraints, so that nations should direct scarce resources to their optimal use. Our model shows that it is worthwhile for a rational acting country (that wants to maximize sporting success) to specialize and thus allocate resources to those sports in which the country has RCA.

In this context, different problem or decision complexes can be emphasized. First, government has to identify which sport is associated with RCA. This requires a permanent evaluation of the specific characteristics of the country—e.g., concerning geographical, economic, or biological determinants [24] with regard to possible advantages. However, this raises the question of adequate measurement methods. Already in the context of trade theory, the suitable measurement of RCA leads to discussions (e.g., [49] or [50]). In this context, it becomes apparent that difficulties exist particularly regarding time and place [50]. Most importantly, it is not certain that comparative advantages are stable over time, as framework conditions can change, so monitoring appears to be necessary. In particular, the question arises whether states can influence the comparative cost advantages through their interventions. If this were the case, the behavior of the other states would also have to be monitored continuously.

Like every model there are some limitations concerning the interpretation: We have to emphasize that our study builds on some typical assumptions for an explanatory approach, such as utility maximization and that history matters within the development of specialization.

Especially, if the sports with relevant RCA are known a priori, government decides in our rational choice model to allocate its scarce resources to this discipline. However, in the reality of sports policy in a democratic state order, further problems open up at this point. These arise in particular from the information incompleteness that exists in reality when decisions are made.

Public Choice literature (of sports) assumes already that political actors are primarily interested in maximizing their votes to stay in office (see [51] or [52]; for sports e.g., [53]). This central characteristic of politicians provides the possibility of rent-seeking to interest groups via targeted lobbying (e.g., [54–57] or [58]). Thus, in the field of organized sports, different sports and their interest groups will try to capture the largest possible share of the distributable budget, which may lead to misallocations under incomplete and asymmetric information [59]. The task of lobbying can be interpreted as to reduce this information gap (e.g., [59] or [60]). From the politician’s point of view, however, it is difficult to verify the truthfulness of information provided by the lobbyist. In practice, each sport will put forward arguments as to why it has RCA and should therefore be given preference in funding. This raises the question of which institutions can ensure that funds are actually allocated to their optimal use according to RCA. To reduce quality uncertainty, a sort can use initial success as a signal for its RCA. As a result, government will allocate more funding in this sport, which will lead to further success, creating a Matthew effect [34].

In addition, a federal state structure is likely to give rise to additional problems, since certain self-governing states may benefit more from certain sports than from others. This can also hamper an optimal allocation of resources to the sports with the corresponding RCA. Moreover, since time lags occur and the (positive effect) of public funding only becomes visible in later periods, there is a risk that political actors will deviate from the decision that is rational according to our model, especially if the election periods differ.

Furthermore, problematic with our approach is that we are not able to test it empirically due to lack of data at the moment. Sports policy data is not always available or there is no differentiation on different disciplines. However, the model can explain what can be observed in the real world and helps to understand the patterns of specialization which is important for policy and science.

## 5 Concluding remarks

International competition in sports is also important from a political perspective, especially for the current governments to build up reputation within the international community of nations. Furthermore, international success in sports could lead to a higher national pride (e.g., [48, 61]) within the population and could therefore enhance a stronger commitment to the country. In scientific literature, there is a long history in determining factors which lead to success in sports. In the beginning of these examinations, the focus was mainly set on geographical differences in order to explain differences in success. Later, also other factors on the macro-level were included and several authors concluded that mainly the GDP (per capita) as well as the size of a population would explain success in international competition of different nations. Similar to athletic accomplishments, the factors which determine specialization patterns in sports were searched on the macro-level. Tcha and Pershin (2003) as well as DuBois and Heyndels (2012) explain specialization in sports with the help of the neoclassical trade model and therefore with the (revealed) comparative advantages in sports ([24, 28]).

In this paper we provide a model to explain the country-related specialization independent of the usual influencing factors. In comparison to other studies our two-country model shows that neither economical nor geographical factors alone determine the specialization in sport, but that specialization is also a matter of political decision-making. With our model, we can explain not only different specialization patterns between, e.g., Europe and Africa but also different specialization patterns within one continent and different patterns of countries with the similar macro-level factors can be understood: Political decisions are able to lead a nation’s sport in a special direction (the so-called “German Democratic Republic” is a good example here). Even when the starting points of two different countries are more or less identical, which is often the case within Western-Europe, our model shows that meso-level factors can lead to different specialization patterns. Our findings can be helpful for sport politicians as well as for sports science. With respect to this, the present analysis implies that governments could build their sports policy on those meso-level factors to find a priori a more or less optimal strategy for their specific country. Moreover, structural changes in sports can be explained as tradition plays a crucial role in sports policy as well.
